# First report of southern root-knot nematode, *Meloidogyne incognita*, infecting pomegranate, *Punica granatum*, in Peru

**DOI:** 10.21307/jofnem-2020-026

**Published:** 2020-04-24

**Authors:** Ricardo Andreé Vega-Callo, María Yaquelin Mendoza-Lima, Nataly Ruth Mamani-Mendoza, Leslie Sharon Lozada-Villanueva, Juan José Tamo-Zegarra, Teodocia Gloria Casa-Ruiz, Cristiano Bellé

**Affiliations:** 1 Universidad Nacional de San Agustin de Arequipa, Arequipa, Peru; 2 Universidade Federal de Santa Maria, Santa Maria, Brazil

**Keywords:** Detection, diagnosis, identification, root-knot nematodes, pomegranate

## Abstract

*Punica granatum* plants showing symptoms caused by root-knot nematodes were detected in the municipality of Majes, Arequipa, Peru. Based on the morphological, esterase phenotypes, and molecular analyses of the mitochondrial DNA region between the cytochome oxidase subunit II and 16 S rRNA genes (mtDNA) and species-specific sequence characterized amplified region, the causal agent of the observed symptoms was identified as *Meloidogyne incognita*. Pathogenicity was confirmed by fulfilling a modified version of Koch’s postulates. To our knowledge, this is the first report of *M. incognita* infecting *P. granatum* in Peru.

Pomegranate (*Punica granatum* L.) is an exotic fruit in Peru that has unique pharmacological characteristics including several bioactive compounds. Its cultivation is intended for ornamentation, fruit production for fresh consumption, or processed products, such as juices, syrups, and jellies (Saroj et al., 2008), among others.

Plants can be attacked by pests, diseases, and plant-parasitic nematodes, which can qualitatively and quantitatively impair production (Dias-Arieira et al., 2010; Sikora et al., 2018). Among the plant-parasitic nematodes, the most important the genus is *Meloidogyne* Göldi, 1887, which causes damage in the form of root galls and reduction in the number of roots, and predisposition to fungal and bacterial diseases causing losses in crop yields (Karssen, 2002; Sikora et al., 2018). Furthermore, root-knot nematodes often thrive and cause damage on perennial hosts for many years preventing them from reaching their full yield potential. The root-knot nematodes, *Meloidogyne incognita* (Kofoid and White, 1919; Chitwood, 1949) and *M. javanica* (Treub, 1885; Chitwood, 1949), the economically important parasites of pomegranate cultivars in the world (Singh et al., 2019).

In a six-year-old pomegranate (cv. Wonderful) plantation aged six years old in Majes, Arequipa, Peru (16°19´37.0˝S; 72°13'08.0"W), plants after pruning were slow to develop new shoots (Figure 1A) and roots with distinct galls (Figure 1B-D) were collected on September, 2019. In order to identify the plant-parasitic nematode species, a combination of morphological, biochemical, and molecular analyses were performed.

**Figure 1:**
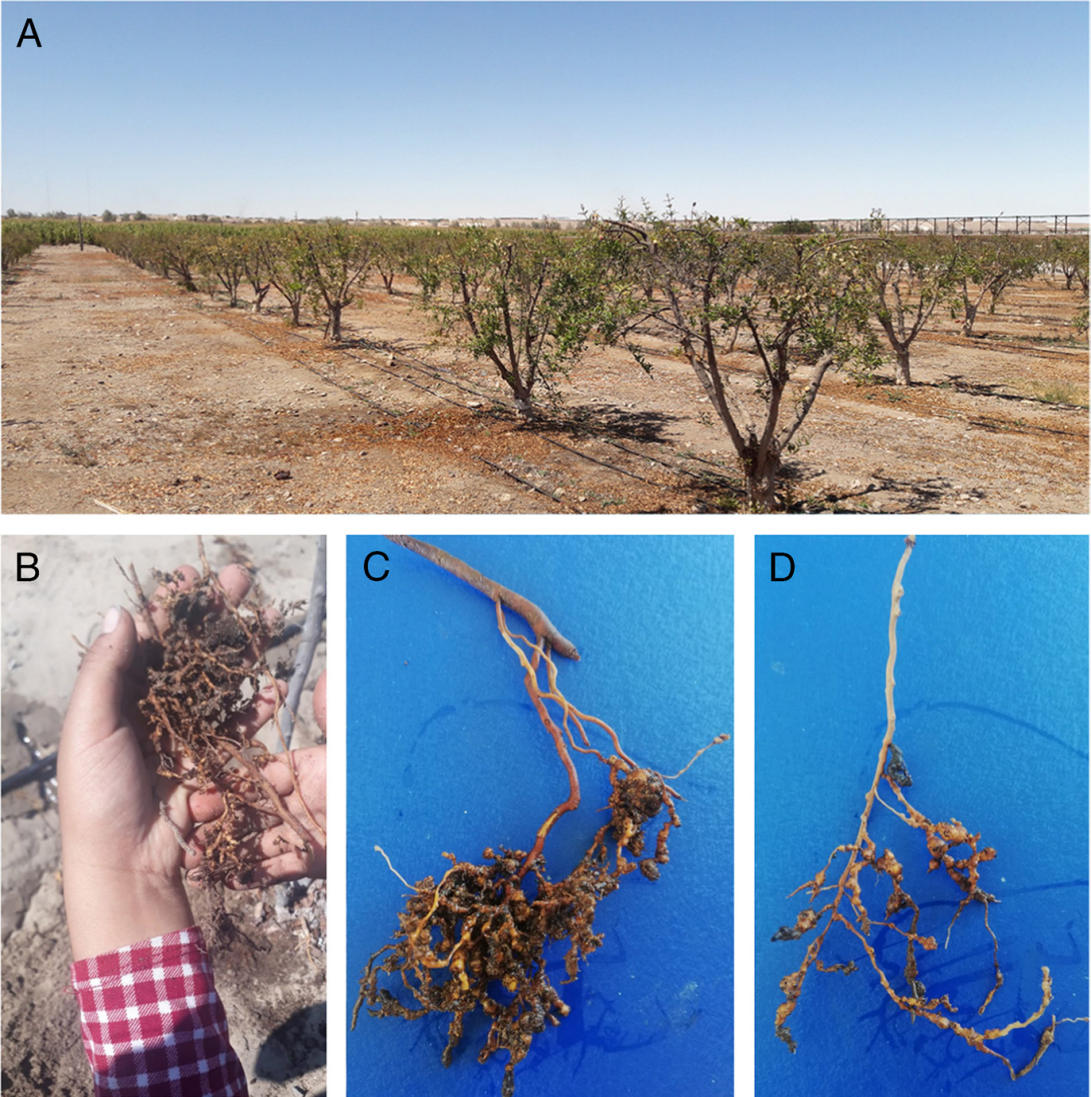
A: Plants of *Punica granatum* L. (cv. Wonderful) plants after pruning, showing the slow development of the plants shoots of the galled root system infected with *Meloidogne incognita* (Kofoid and White, 1919; Chitwood, 1949). B-D: Closeup view of the root system of the *P*. *granatum* infected with *M. incognita* showing galls in roots.

This population of root-knot nematode was identified to species with esterase phenotypes (*n* = 36 females) (Carneiro and Almeida, 2001); morphology and morphometrics of second-stage juveniles (J2) (*n* = 30) and females (*n* = 10), and perineal patterns (*n* = 15); and molecular characterization of the mitochondrial DNA region between the cytochome oxidase subunit II (COII) and 16 S rRNA genes (mtDNA) using the primers C2F3 (5´-GGTCAATGTTCAGAAATTTGTGG-3´) and 1108 (5´-TACCTTTGACCAATCACGCT-3´) (Powers and Harris, 1993) along with PCR species-specific sequence characterized amplified region (SCAR) for confirmation, using a primer set composed of inc-K14-F (5´-GGGATGTGTAAATGCTCCTG-3´) and inc-K14-R(5´-CCCGCTACACCCTCAACTTC-3´) (Randig et al., 2002).

The nematode population density was 1,500 second-stage juveniles (J2)/g of root. Morphometric study showed the following results; J2s: length (L) = 350.5 ± 25.7 (315-490) μm, a = 23.0 ± 4.5 (20.1-26.5), c = 8.9 ± 0.9 (5.0-10.5), stylet length = 11.5 ± 0.5 (9.2-12.4) μm, dorsal esophageal gland orifice to base of stylet (DGO) = 2.4 ± 0.3 (1.8-2.9) μm, tail length = 40.5 ± 1.0 (39.0-48.5) μm and hyaline tail terminus = 10.3 ± 0.8 (10.1-11.2) μm. Morphometrics of females: L = 645.5 ± 30.0 (544.5-705.5) μm, stylet length = 14.2 ± 0.5 (12.4-15.7) μm, and DGO = 3.6 ± 0.2 (2.9-4.1) μm. The perineal pattern of the female included a high and square dorsal arch with wavy striae bending toward the lateral lines and the absence of distinct lateral line incisures (Figure 2A-C). The overall morphology and morphometrics of this population appears similar to that of *M. incognita* (Hunt and Handoo, 2009).

**Figure 2:**
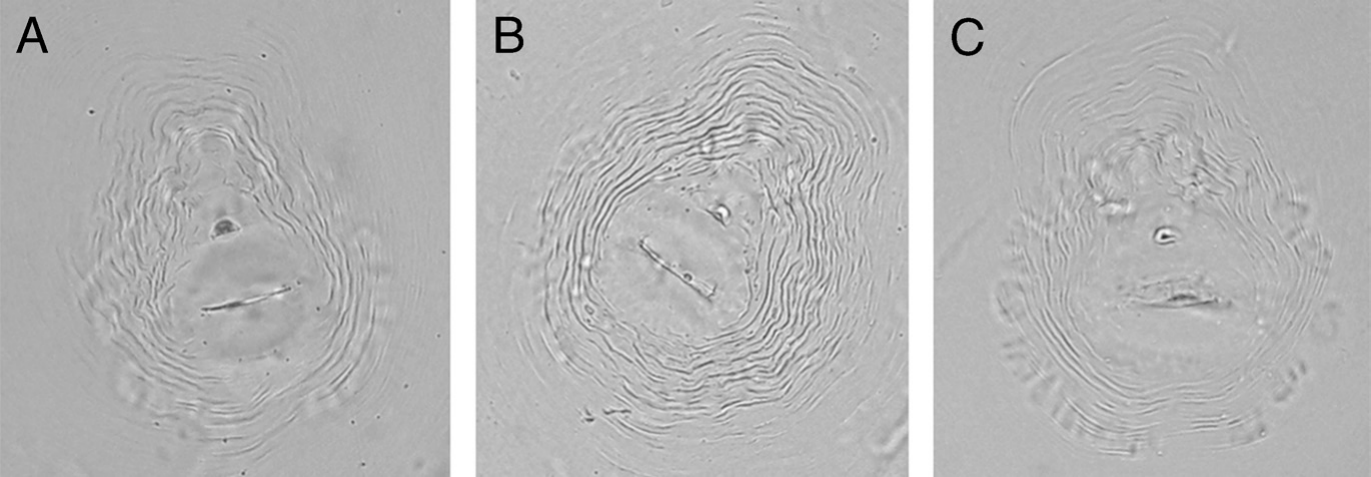
A-C: *Meloidogyne incognita* (Kofoid and White, 1919; Chitwood, 1949) perineal patterns detected in *Punica granatum* L. (cv. Wonderful) (Scale for light microscopy photos = 10 μm).

The polymorphisms of the esterase bands by electrophoresis revealed the phenotype I2 (Rm = 1.05 and 1.10) typical of *M. incognita* (Carneiro et al., 1996). The mtDNA sequence (1,638 bp) was submitted to GenBank with Accession No. MT066217.1. Searches on BLAST showed a 99% identity with sequences of *M. incognita* isolates from Brazil (GenBank MK861920.1), USA (GenBank KP001567.1 and KF993635.1), and China (GenBank MH152335.1 and MH152333.1). The PCR amplification using SCAR technique produced a specific fragment of expected size (∼399 bp) for *M. incognita* (Randig et al., 2002).

In greenhouse tests, *P. granatum* (cv. Wonderful) plantlets were maintained in pots with 5,000 dm^3^ sterilized soil. In total, eight replicates were inoculated with 5,000 eggs and J2s from the original population of *M. incognita*, in addition to a non-inoculated control. Plants were well maintained under greenhouse conditions at 25 ± 3°C. After 120 days, the inoculated plants exhibited galled root systems similar to plants observed in the field, with a nematode reproduction factor (final population/initial population) of 18.5. The non-inoculated plants did not exhibit any galls. The morphological and molecular characterization of this re-isolated root-knot nematode were identical those of *M. incognita*.

This is the first report of *M. incognita* parasitizing pomegranate plants in Peru. This finding has great importance for the fruit, and nursery industry in Peru, since this nematode may damage pomegranate plants and become more widespread and a significant problem for this crop.
